# Bipolar disorder correlated to shorter remission latency and borderline personality disorder symptom severity to longer in depression – a prospective cohort study of major depressive patients

**DOI:** 10.1192/j.eurpsy.2022.257

**Published:** 2022-09-01

**Authors:** J. Söderholm, L. Socada, T. Rosenström, J. Ekelund, E. Isometsä

**Affiliations:** 1 University of Helsinki, Department Of Psychiatry, University of Helsinki, Finland; 2 University of Helsinki, Department Of Psychology And Logopedics, University of Helsinki, Finland; 3 Hospital District of Helsinki and Uusimaa, Department Of Psychiatry, HUS, Finland

**Keywords:** bipolar disorder, outcome, Depression, borderline personality disorder

## Abstract

**Introduction:**

Major depressive episodes (MDE) occur in major depressive (MDD) and bipolar disorders (BD), and are frequently complicated by borderline personality disorder (BPD). Mixed affective symptomatology is a hallmark of BD, and affective lability of BPD; both may markedly influence illness course. However, direct comparisons of outcome of depression in MDD, BD and BPD are scarce.

**Objectives:**

To investigate course of illness and outcome of depression in MDD, bipolar and borderline patients.

**Methods:**

In this six-month, prospective cohort study of secondary-level psychiatric MDE patients (n = 95), after initial assessment, the patients (N = 95) completed biweekly online assessments of mood symptoms. We divided the follow up period into qualitatively different mood state periods based on multiple prospective information sources. We examined mixed affective symptoms and borderline symptom severity dimensionally. Outcomes assessed included clinical course, time to first full symptomatic remission, and factors predicting these.

**Results:**

Remission rates according to DSM-5 were similar in MDD, MDE/BD and MDE/BPD patients. Bipolar patients experienced more shorter qualitatively distinct mood state periods during follow-up than the others. Bipolar disorder was associated with shorter (HR = 2.44, 95% CI = 1.27–4.67, see fig. 1) and dimensionally assessed BPD severity with longer time to first remission (HR = 0.95 per point., CI = 0.91–1.00).

**Figure:**
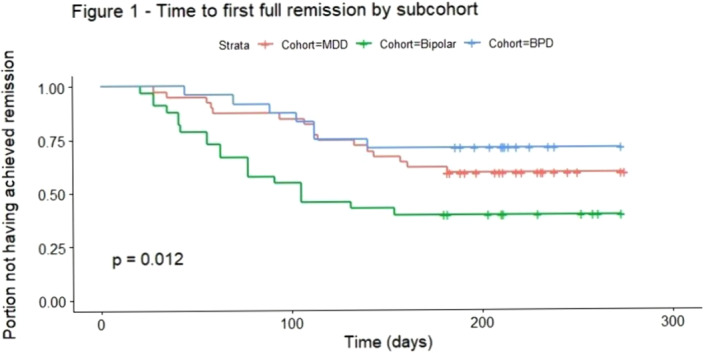

**Conclusions:**

Course of illness differs between the three depressive groups in the medium term. Bipolar depressive patients have the most alternating course and the shortest time to first remission. Dimensionally assessed severity of BPD may be prognostic of longer depressive remission latency.

**Disclosure:**

I am employed by a psychiatric treatment provider, treating e.g. patients suffering from depression, bipolar disorder and borderline personality disorder.

